# Associations of insulin resistance, sex hormone-binding globulin, triglyceride, and hormonal profiles in polycystic ovary syndrome: A cross-sectional study

**DOI:** 10.18502/ijrm.v19i7.9476

**Published:** 2021-08-16

**Authors:** Bahia Namavar Jahromi, Niloofar Borzou, Mohammad Ebrahim Parsanezhad, Zahra Anvar, Parvin Ghaemmaghami, Soudabeh Sabetian

**Affiliations:** ^1^Infertility Research Center, Shiraz University of Medical Sciences, Shiraz, Iran.; ^2^Department of Obstetrics and Gynecology, School of Medicine, Shiraz University of Medical Sciences, Shiraz, Iran.; ^3^Student Research Center, Shiraz University of Medical Sciences, Shiraz, Iran.; ^4^School of Nursing and Midwifery, Shiraz University of Medical Sciences, Shiraz, Iran.

**Keywords:** Polycystic ovary syndrome, Insulin resistance, Sex hormone-binding globulin.

## Abstract

**Background:**

Insulin resistance (IR) occurs in 50–70% of women with polycystic ovary syndrome (PCOS) and can be applied as a prediabetic feature in PCOS.

**Objective:**

In this study, indirect methods including fasting blood sugar (FBS), fasting insulin (FI), FBS/FI ratio, and quantitative insulin sensitivity check index (QUICKI) were compared with the homeostasis model assessment of insulin resistance (HOMA-IR) as a standard technique. The association of IR to sex hormone-binding globulin (SHBG) and several hormones was also analyzed.

**Materials and Methods:**

This cross-sectional study was performed on 74 PCOS women. Sensitivity and specificity of each IR method was calculated based on HOMA-IR. Hormonal profiles of the patients were compared between the groups with defined normal and abnormal values of IR.

**Results:**

Triglyceride levels had a positive association with FBS and HOMA-IR (p = 0.002 and p = 0.01, respectively) with a negative association to QUICKI and SHBG (p = 0.02 and p = 0.02, respectively). SHBG showed a significant negative association with FBS (p = 0.001). Dehydroepiandrosterone sulfate showed a positive association with FI (p = 0.002). Seven PCOS women showed abnormal SHBG levels (< 36 nmol/L) while expressed normal values of the rest of the studied variables. FI and QUICKI had the highest sensitivity while FBS/FI and QUICKI had the highest specificity when HOMA-IR was applied as a standard test.

**Conclusion:**

SHBG and triglyceride had a significant negative and positive association with IR, respectively. HOMA-IR followed by FI and QUICKI is the most sensitive test for the detection of IR. SHBG levels can be a helpful biomarker for the diagnosis of PCOS.

## 1. Introduction

Polycystic ovary syndrome (PCOS) classified as World Health Organization (WHO) group II ovulation disorders is one of the most common health problems in women of reproductive age recognized by chronic anovulation (1). PCOS is associated with insulin resistance (IR), hyperinsulinemia, and obesity. IR is detected in 50–70% of PCOS women with normal body mass index (BMI), whereas obese PCOS women show a higher prevalence of IR (2). IR along with high insulin levels stimulates the ovaries to produce more androgens. IR in PCOS increases the risk of diabetes and prediabetic states (3). About 60% of PCOS women exhibit high levels of serum androgens including testosterone, androstenedione, and dehydroepiandrosterone sulfate (DHEAS) (4). Low levels of sex hormone-binding globulin (SHBG) were reported to be associated with obesity, IR, hyperandrogenism, glucose intolerance, and type-2 diabetes in women with PCOS. It was observed that the therapeutic balance of SHBG can improve PCOS-dependent morbidities (5). Therefore, we hypothesized that SHBG levels might be a helpful biomarker for the diagnosis and treatment of PCOS or IR. Modifications of lifestyle and diet according to the medical guidelines and preventive strategies in general practice is the first line of treatment for PCOS (6). A proper prediabetes screening test is important to be performed for all PCOS women (7). On the other hand, IR can be estimated by various methods ranging from complex to simple techniques with different accuracies (8). To perform a standard method like hyperinsulinemic-euglycemic clamp (HEC) or the frequently sampled intravenous glucose tolerance test (FSIVGTT), multiple blood samples are collected (9). Due to the complexity of HEC and FSIVGTT, simple and semi-invasive methods are preferred for the clinical settings (10). Fasting blood sugar (FBS) and fasting insulin (FI) levels are suggested to detect prediabetic states in PCOS patients (11). FBS-to-FI ratio (FBS/FI), homeostasis model assessment of insulin resistance (HOMA-IR), and the quantitative insulin sensitivity check index (QUICKI) are important indicators of insulin sensitivity for patients with hyperinsulinemia (12). Although several tests are introduced to detect IR, no one could obtain general public for both clinical and research purposes (13).

It was previously reported that IR and lipid profiles are positively correlated with total testosterone and free androgenic index (14). Also, triglyceride (TG) was suggested to be considered as a valuable substitute marker for IR in women with PCOS (15). In this research, we studied the relationship between IR, SHBG, TG, and hormonal profiles of PCOS women including the sensitivity, specificity, positive predictive value (PPV), and negative predictive value (NPV) of each IR method calculated based on HOMA-IR.

## 2. Materials and Methods

### Study design

This cross-sectional study included 74 PCOS women diagnosed according to the Modified Rotterdam Criteria 2003 (16). In order to diagnose PCOS, two of the following criteria are required: (i) menstrual abnormalities (amenorrhea, oligomenorrhea), (ii) clinical and/or biochemical hyperandrogenism, and (iii) the ultrasound look of polycystic ovaries. Additionally, it is important to exclude other disorders such as Cushing's syndrome, congenital adrenal hyperplasia, and androgen-secreting tumors which have similar clinical presentation to PCOS.

### Inclusion and exclusion criteria

PCOS women referred to the clinics affiliated to Shiraz University of Medical Sciences from January to December 2018, aged 18–48 yr, and willing to participate were enrolled in this study. The exclusion criteria were pregnancy, lactation, hypertension, and diabetes. The patients who took insulin-sensitizing agents, hormonal treatments, or corticosteroids in the past two months prior to the study were not included.

### Sample collection and outcome measurement 

Blood samples were collected from PCOS women after an overnight fasting for 10–12 hr. Serum was extracted by centrifugation at 3000 g for 20 min and stored at –20∘C. Luteinizing hormone (LH), follicle-stimulating hormone (FSH), thyroid-stimulating hormone (TSH), and prolactin and testosterone levels were measured by immune radiometric assay (RIA kit IRMA tube, Korea). SHBG and DHEAS levels were measured by radioimmunoassay (Testo-RIA kit, France) and TG by ELISA (BioVendor ELISA kit, Germany). FI and FBS values were checked by immunoradiometric assay (IRMA kit, Hungary).

In this project, the following five indirect methods were employed to assess IR in PCOS patients: FBS, FI, FBS/FI ratio, HOMA-IR, and QUICKI. We used the following formulas: HOMA = FI (µ U/mL) × fasting glucose (mg/dL)/405 and QUICKI = 1/ (log (FI µU/mL) + log (fasting glucose mg/dL)). FBS ≥ 100 (mg/dl), FI ≥ 10 (uIU/mL), FBS/FI < 4.5, HOMA-IR ≥ 2.5, and QUICKI ≤ 0.33 were considered as the cut-off values in favor of IR (17, 18). BMI < 25 was considered normal. IR values were compared between the patients with BMI ≥ 25 and those with < 25. Also, SHBG ≥ 36 (nmol/L) was considered normal and data were compared between women with SHBG ≥ 36 and SHBG < 36 (nmol/L) (19). The values of hormones and TG were also compared between the mentioned groups. Previous studies showed that the results of HOMA-IR assessment were correlated significantly with the results of the clamp studies (20). Also, HOMA-IR had the highest sensitivity according to recent research (18). Therefore, we substituted HOMA-IR for the standard technique and compared the sensitivity, specificity, PPV, and NPV of the other methods to HOMA-IR.

### Ethical considerations

All participants signed a written informed consent form before enrollment and after a complete explanation of the study design. The Medical Ethics Committee of Shiraz University of Medical Sciences approved the study protocol (Code: IR.SUMS.REC.1394.S612).

### Statistical analysis

In this cross-sectional study, 74 PCOS women were enrolled by simple random sampling. Data are presented as mean ± SD and were analyzed using SPSS software v. 19.0 (Statistical Package for the Social Sciences software, version 19.0, SPSS Inc., Chicago, IL, USA). *T* tests were used to compare the study parameters. P < 0.05 was considered significant. The predictive ability of covariates (with p-value < 0.25 in *t* tests) on IR (HOMA-IR ≥ 2.5) was evaluated using logistic regression analysis.

## 3. Results

The demographic, clinical, and laboratory characteristics of the enrolled PCOS women are presented in Table I.

In this study, no significant correlation was observed between the BMI and IR in the PCOS patients. Table II presents a comparison of the hormonal profiles according to BMI and five indirect methods of IR.

TG was significantly higher in women with abnormal FBS (p = 0.002), QUICKI (p = 0.02), or HOMA-IR (p = 0.01). DHEAS was significantly higher among those with abnormal FI (p = 0.002). SHBG showed a significant negative association with FBS (p = 0.001).

HOMA-IR was applied as the standard test to determine IR. Table III shows the results of logistic regression analysis assessing the effectiveness of the covariates (with p-value < 0.25 in *t* tests) including testosterone, SHBG, and TG in predicting the outcome of IR (if HOMA-IR ≥ 2.5 indicate IR). The results represented that the mentioned covariates were not significantly predictive of IR.

The measured variables and demographic data were classified according to the normal and abnormal SHBG levels, as presented in Table IV.

SHBG showed a significant negative association with TG (p = 0.02). The PCOS women with SHBG ≥ 36 (nmol/L) had statistically significant lower TG and higher FSH levels (p < 0.5). There were seven PCOS women with SHBG < 36 nmol/L, while all of the other measured variables were in the normal ranges.

We compared the sensitivity, specificity, PPV, and NPV of FBS, FI, FBS/FI, and QUICKI with that of HOMA-IR (Table V).

**Table 1:** Clinical and laboratory characteristics of the PCOS women


**Variable**	**Mean ± SD**	**95% CI of the difference**
**Age (yr)**	27.31 ± 5.01	26.33–28.28
**BMI (kg/m${}^{2}$)**	26.37 ± 5.21	25.16–27.57
**FBS (mg/dl)**	90.02 ± 7.83	88.21–91.84
**FI (uIU/mL)**	13.60 ± 12.59	10.68–16.52
**FSH (IU/L)**	5.57 ± 2.28	5.04–6.10
**TSH (mU/L)**	2.88 ± 3.26 (1.64^♦^)	2.12–3.64
**LH (IU/L)**	8.64 ± 6.80	7.03–10.25
**Prolactin (ng/mL)**	11.57 ± 7.44	9.80–13.35
**SHBG (nmol/L)**	30.80 ± 20.79	25.95–35.65
**Testosterone (ng/mL)**	1.22 ± 1.89 (0.4^♦^)	0.77–1.67
**DHEAS (μg/dL)**	200.56 ± 141.37	167.57–233.54
**TG (mg/dL)**	119.04 ± 67.45	102.33–135.76
BMI: Body mass index, SD: Standard deviation, FBS: Fasting blood sugar, FI: Fasting insulin, FSH: Follicle-stimulating hormone, TSH: Thyroid-stimulating hormone, LH: Luteinizing hormone, SHBG: Sex hormone-binding globulin, DHEAS: Dehydroepiandrosterone sulfate, TG: Triglyceride, CI: Confidence interval. ^♦^IQR: Interquartile range		

**Table 2:** Hormonal profiles according to normal and abnormal values of BMI, FBS, FI, HOMA-IR, QUICKI, and FBS/FI


**Variables**	**FSH (IU/L)**	**LH (IU/L)**	**TSH (mU/L)**	**Testosterone (ng/mL)**	**DHEAS (μg/dL)**	**PRL (ng/mL)**	**SHBG (nmol/L)**	**TG (mg/dL)**
**BMI** **(kg/m${}^{2}$)**								
**< 25**	5.92 ± 2.71	8.35 ± 5.43	3.26 ± 4.49 (1.71^♦^)	1.00 ± 1.14 (0.47^♦^)	206.70 ± 144.60	13.36 ± 8.19	30.84 ± 23.87	121.70 ± 77.26
**≥ 25**	5.27 ± 1.82	8.90 ± 7.88	2.54 ± 1.39	1.42 ± 2.38 (0.45^♦^)	194.91 ± 140.03	10.07 ± 6.48	30.77 ± 18.00	116.77 ± 58.83
**p-value**	0.23	0.73	0.35	0.35	0.72	0.065	0.99	0.77
**FBS (mg/dl)**								
**< 100**	6.09 ± 5.51	8.72 ± 7.12	3.26 ± 4.17 (2.5^♦^)	1.37 ± 2.08 (0.4^♦^)	192.37 ± 126.05	11.76 ± 8.14	34.25 ± 21.79	111.39 ± 62.87
**≥ 100**	5.59 ± 2.12	8.84 ± 6.22	2.12 ± 0.93	0.64 ± 0.21	186.71 ± 176.09	10.75 ± 2.81	19.68 ± 9.72	171.66 ± 77.56
**p-value**	0.69	0.95	0.23	0.1	0.87	0.42	0.001*	0.002*
**FI** **(uIU/mL)**								
**< 10**	6.71 ± 7.57 (3.34^♦^)	9.50 ± 8.82	2.74 ± 1.37	1.43 ± 2.53 (0.47^♦^)	139.32 ± 92.44	11.99 ± 7.67	33.90 ± 18.81	103.17 ± 43.64
**≥ 10**	5.49 ± 2.28	8.43 ± 5.63	3.23 ± 4.74 (1.45^♦^)	1.08 ± 1.33 (0.3^♦^)	219.63 ± 150.41	11.27 ± 7.36	28.57 ± 21.46	130.32 ± 77.10
**p-value**	0.26	0.49	0.47	0.5	0.002*	0.7	0.22	0.05*
**HOMA-IR**								
**< 2.5**	6.35 ± 6.73 (3.25^♦^)	9.14 ± 8.28	2.64 ± 1.35	1.68 ± 2.61 (0.57^♦^)	163.94 ± 118.83	10.95 ± 7.66	34.56 ± 23.06	100.79 ± 46.26
**≥ 2.5**	5.58 ± 2.34	8.53 ± 5.47	3.48 ± 5.29 (1.55^♦^)	0.8 ± 0.58	214.36 ± 149.43	12.15 ± 7.28	26.50 ± 16.84	140.36 ± 80.15
**p-value**	0.46	0.68	0.31	0.06	0.50	0.50	0.06	0.01*
**QUICKI**								
**> 0.33**	6.60 ± 7.13 (3.32^♦^)	9.72 ± 8.65	2.66 ± 1.33	1.68 ± 2.61 (0.57^♦^)	165.69 ± 125.08	10.95 ± 7.66	32.46 ± 17.73	100.75 ± 45.67
**≤ 0.33**	5.46 ± 2.32	8.15 ± 5.37	3.34 ± 4.94 (1.55^♦^)	0.80 ± 0.58	206.31 ± 142.96	12.15 ± 7.28	29.19 ± 22.55	135.11 ± 77.85
**p-value**	0.28	0.30	0.34	0.06	0.15	0.50	0.45	0.02*
**FBS/FI**								
**≥ 4.5**	6.08 ± 5.38	9.11 ± 7.33	3.08 ± 4.02 (1.43^♦^)	1.31 ± 2.01 (0.4^♦^)	186.98 ± 140.8	10.96 ± 6.78	31.99 ± 20.98	115.11 ± 66.05
**< 4.5**	5.23 ± 1.85	7.11 ± 4.10	2.76 ± 1.75	0.60 ± 0.13	198.81 ± 97.95	15.70 ± 10.54	20.23 ± 13.45	157.70 ± 72.85
**p-value**	0.59	0.36	0.79	0.29	0.79	0.07	0.07	0.06
BMI: Body mass index, FBS: Fasting blood sugar, FI: Fasting insulin, FSH: Follicle-stimulating hormone, TSH: Thyroid-stimulating hormone, LH: Luteinizing hormone, SHBG: Sex hormone-binding globulin, DHEAS: Dehydroepiandrosterone sulfate, PRL: Prolactin, TG: Triglyceride, HOMA-IR: Homeostasis model assessment of insulin resistance, QUICKI: Quantitative insulin-sensitivity check index. All data are presented as Mean ± SD. ${}^{*}$P-value < 0.05 is considered significantly different using independent sample *t* test. ^♦^IQR: interquartile range								

**Table 3:** Logistic regression analysis for HOMA-IR


**Variables**	**Coef**	**SE Coef**	**p-value**	**OR (95% CI)**
**Testosterone (ng/mL)**	–0.286	0.201	0.15	0.75 (0.51–1.11)
**SHBG (nmol/L)**	–0.002	0.013	0.86	0.99 (0.97–1.02)
**TG (mg/dL)**	0.005	0.005	0.25	1.01 (0.99–1.01)
**Constant**	–0.136	0.845	0.87	–
SHBG: Sex hormone-binding globulin, TG: Triglyceride, Coef: Coefficient, SE Coef: Standard error of the coefficient, OR: Odds ratio, CI: Confidence interval. ${}^{*}$P-value < 0.05 is considered significantly different using logistic regression analysis				

**Table 4:** The comparison of demographic data, hormonal profile, and IR in PCOS patients classified based on SHBG levels


**Variables**	**SHBG ≥ 36 (nmol/L)**	**SHBG < 36 (nmol/L)**	**p-value**	**95% CI of the difference**
**Age (yr)**	25.81 ± 5.07	27.71 ± 4.91	0.08	–4.03–0.23
**BMI (kg/m${}^{2}$)**	25.89 ± 4.65	26.66 ± 5.45	0.57	–3.47–1.92
**FSH (mIU/mL)**	6.50 ± 2.97	5.19 ± 1.89	0.03*	0.14–2.48
**TSH (mU/L)**	2.32 ± 1.67	3.10 ± 3.74 (1.61^♦^)	0.36	–2.48–0.91
**LH (IU/L)**	8.95 ± 5.41	8.53 ± 7.33	0.82	–3.19–4.03
**DHEAS (μg/dl)**	194.68 ± 145.18	199.24 ± 140.23	0.90	–78.87–69.74
**Testosterone (ng/ml)**	2.17 ± 3.17 (0.52^♦^)	0.88 ± 0.95 (0.4^♦^)	0.09	–0.25–2.84
**Prolactin (ng/mL)**	11.34 ± 8.96	11.71 ± 6.95	0.85	–4.42–3.69
**Triglyceride (mg/dl)**	92.56 ± 40.93	129.06 ± 72.46	0.02*	–65.94–7.06
**FBS (mg/dL)**	87.71 ± 7.38	91.06 ± 7.92	0.10	–7.35–0.66
**FBS/FI**	12.40 ± 8.60	9.96 ± 7.64	0.24	–1.65–6.51
**FI (uIU/mL)**	12.56 ± 16.67 (8.12^♦^)	14.11 ± 10.82	0.64	–8.12–5.02
**HOMA**	2.70 ± 3.41 (1.72^♦^)	3.20 ± 2.59	0.49	–1.97–0.96
**QUICKI**	0.35 ± 0.04	0.33 ± 0.03	0.11	–0.003–0.03
SHBG: Sex hormone-binding globulin, BMI: Body mass index, FSH: Follicle-stimulating hormone, TSH: Thyroid-stimulating hormone, LH: Luteinizing hormone, DHEAS: Dehydroepiandrosterone sulfate, FBS: Fasting blood sugar, FI: Fasting insulin, HOMA-IR: Homeostasis model assessment of insulin resistance, QUICKI: Quantitative insulin-sensitivity check index, CI: Confidence interval. All data are presented as Mean ± SD. ${}^{*}$P-value < 0.05 is considered significantly different using independent sample *t *test. ^♦^IQR: Interquartile range				

**Table 5:** Sensitivity, specificity, PPV, and NPV of the five methods for detection of IR based on HOMA-IR


	**FBS ≥ 100**	**FI ≥ 10**	**FBS/FI < 4.5**	**QUICKI ≤ 0.33**	**HOMA-IR ≥ 2.5**
Insulin resistant*	14 (19%)	45 (61%)	9 (12%)	38 (51%)	38 (51%)
Non-insulin resistant *	60 (81%)	29 (39%)	65 (88%)	36 (49%)	36 (49%)
Total*	74 (100%)	74 (100%)	74 (100%)	74 (100%)	74 (100%)
Sensitivity**	26%	100%	24%	100%	–
Specificity **	88%	81%	100%	100%	–
PPV**	71.4%	84%	100%	100%	–
NPV**	53%	100%	55%	100%	–
*Data presented as n (%). **Data presented as percentages. HOMA-IR: Homeostasis model assessment of insulin resistance, FBS: Fasting blood sugar, FI: Fasting insulin, QUICKI: Quantitative insulin-sensitivity check index, PPV: Positive predictive value, NPV: Negative predictive value					

## 4. Discussion

In this research, 74 PCOS women were studied for their serum SHBG, hormonal profiles, TG levels, and five indirect methods for the detection of IR. They were classified according to their normal and abnormal BMI, IR, and SHBG levels, and data were compared to detect any possible relationships. Also, sensitivity, specificity, PPV, and NPV of the five indirect methods of IR were calculated based on the HOMA-IR test.

Previous studies have shown BMI to be positively associated with IR and diabetes (21, 22). However, it has been showed that IR was not associated with waist circumference or BMI (23). Logically, we expected PCOS women with higher BMI to represent more IR. In this study, we observed higher mean levels of FBS, FI, and more abnormal IR values among women with higher BMI but the differences were not statistically significant.

DHEAS is known as a general marker to diagnose extra amounts of adrenal precursor androgen (APA) in PCOS patients (24). Among the PCOS subjects of this study, we detected a significant association between DHEAS and FI levels (p = 0.002). We concluded that the higher levels of insulin could stimulate the production of APA leading to the increased levels of DHEAS.

We found that serum TG levels and IR have positive independent associations, so we think that the evaluation of TG might serve as a useful clinical biomarker to predict IR for PCOS patients to confirm previous reports (15). The results of our study are in good agreement with the previous studies showing TG has a significant correlation with IR detected by the indirect methods of QUICKI (p = 0.02), HOMA-IR (p = 0.01), and FBS (p = 0.002). On the other hand, lower SHBG levels were significantly associated with higher serum TG levels (p = 0.016) and FBS (p = 0.001) in our study. We know that SHBG binds the androgens with a high affinity to regulate the free sex hormones (25). Low levels of SHBG are associated with higher levels of free androgens that can manifest with hirsutism, acne, or irregular menstruations in PCOS patients (26). Although the specific role of SHBG in the glucose metabolism is not clear yet, recent studies have implicated that the alterations in normal sex steroids physiology may have a role in the glucose homeostasis and low SHBG levels may precede the development of type-2 diabetes mellitus (27). In our study, PCOS women with lower SHBG levels had higher mean FBS and TG levels, which is in good consistency with the hypothesis of the relationship between low SHBG with metabolic syndrome and abnormal glucose metabolism. SHBG ≥ 36 nmol/L was considered normal in this project (19). Out of the 74 PCOS patients enrolled in this study, 52 showed SHBG < 36 nmol/L. Interestingly, only seven PCOS women with normal IR, testosterone, and BMI showed abnormal SHBG < 36 nmol/L. The achieved results indicate that the measurement of SHBG might be a helpful biomarker for the diagnosis of PCOS and IR.

IR and hyperandrogenism play important roles in the metabolic features of PCOS and increase the risk of prediabetes state (28). PCOS women are prone to develop type-2 diabetes if ascertained lifestyle modifications are not adopted (29). Consistently, a balanced diet, weight loss, physical activity, and medications improve IR as well as prediabetes state (30). The American Diabetes Association (ADA) suggests an effective lifestyle modification to prevent type-2 diabetes for women who are diagnosed with prediabetes states (31). As IR affects a high percentage of PCOS women with no clear symptoms, a clinically easy-to-perform and practical test with high accuracy and sensitivity is required for earlier diagnosis of prediabetes conditions.

In this project, we compared the sensitivity, specificity, PPV, and NPV of the five indirect methods of IR and found that HOMA-IR, FI, and QUICKI had the highest NPV and sensitivity to reflect the potential prediabetes in PCOS women. In addition, HOMA-IR, QUICKI, and FBS/FI had the highest PPV and specificity. We believe that the performance of a test with a higher sensitivity helps in earlier diagnosis of prediabetes state and this knowledge accompanied by lifestyle changes protects the PCOS women from diabetes and other subsequent complications. We admit that absence of data from non-PCOS women, as a control group, is a limitation of this study design. In addition, it was not possible for us to perform HEC and FSIVGTT direct methods to detect accurate IR for comparison because of the complexity of the procedures.

## 5. Conclusion

SHBG had a significant negative association with FBS and TG. DHEAS displayed a significant positive association with FI. TG represented a strong positive relationship with HOMA-IR and FBS and a significant negative relationship with QUICKI and SHBG levels. Some PCOS patients expressed abnormal SHBG despite normal values of other studied variables. SHBG is speculated to be a potential biomarker to diagnose PCOS. FI and QUICKI had the highest NPV and sensitivity while FBS/FI and QUICKI had the highest PPV and specificity when considering HOMA-IR as a standard test.

##  Conflict of Interest

The authors declare that they have no conflict of interest.
